# Isolated massive histiocytes renal interstitial infiltration: a case report of an unexpected cause of acute kidney injury in a kidney transplant recipient

**DOI:** 10.1186/s12882-023-03135-z

**Published:** 2023-03-28

**Authors:** Luis E. M. Martins, Miguel Moyses-Neto, Roberto S. Costa, Fabiola Traina, Elen A. Romao

**Affiliations:** 1 Division of Nephrology, Ribeirao Preto Medical School, University of Sao Paulo, Ribeirao Preto, Brazil; 2 Department of Medical Imaging, Hematology, and Clinical Oncology, Ribeirao Preto Medical School, University of Sao Paulo, Ribeirao Preto, Brazil

**Keywords:** Acute kidney injury, Graft loss, Histiocytes renal interstitial infiltration, Kidney transplantation, Renal biopsy, Case report

## Abstract

**Background:**

Acute kidney injury is a frequent cause of hospital readmission in kidney transplant recipients (KTR), usually associated with infections and graft rejection. Herein, we report a case of an unusual cause of acute kidney injury in a KTR (massive histiocytes renal interstitial infiltration).

**Case presentation:**

A 40-year-old woman was submitted to a second kidney transplant. One year after surgery, she presented asthenia, myalgia, and fever, haemoglobin 6.1 g/dL; neutrophils: 1.3 × 109/µL; platelets: 143 × 109/µL; blood creatinine 11.8 mg/dL, requiring dialysis. A kidney biopsy revealed diffuse histiocytic infiltration, which was assumed due to dysregulated immunological activation triggered by infections. The patient had multiple infections, including cytomegalovirus infection (CMV), aspergillosis, bacteraemia, and urinary tract infections, which could trigger the immune response. Haemophagocytic lymphohistiocytosis (HLH) was ruled out. The present case highlights the occurrence of isolated massive renal interstitial infiltration of histiocytes that does not meet the criteria for HLH or other related pathologies.

**Conclusions:**

Renal histiocyte activation and infiltration may have been initiated by an immunological mechanism similar to what occurs in HLH and infectious processes. The present case highlights the occurrence of isolated massive renal interstitial infiltration of histiocytes that does not meet the criteria for HLH or other related pathologies.

## Background

Acute kidney injury (AKI) is a frequent cause of hospital readmission in kidney transplant recipients (KTR), usually associated with infections and graft rejection [1, 2]. KTR are more susceptible to developing AKI as a consequence of urinary tract infections, nephrotoxic drugs, and immune-mediated injury because they have a decreased renal “reserve” due to a reduced mass of nephrons. [1, 2]. Herein, we report a case of an unusual cause of acute kidney injury in a KTR (massive isolated histiocytes renal interstitial infiltration).

## Case presentation

**Fig. 1:**
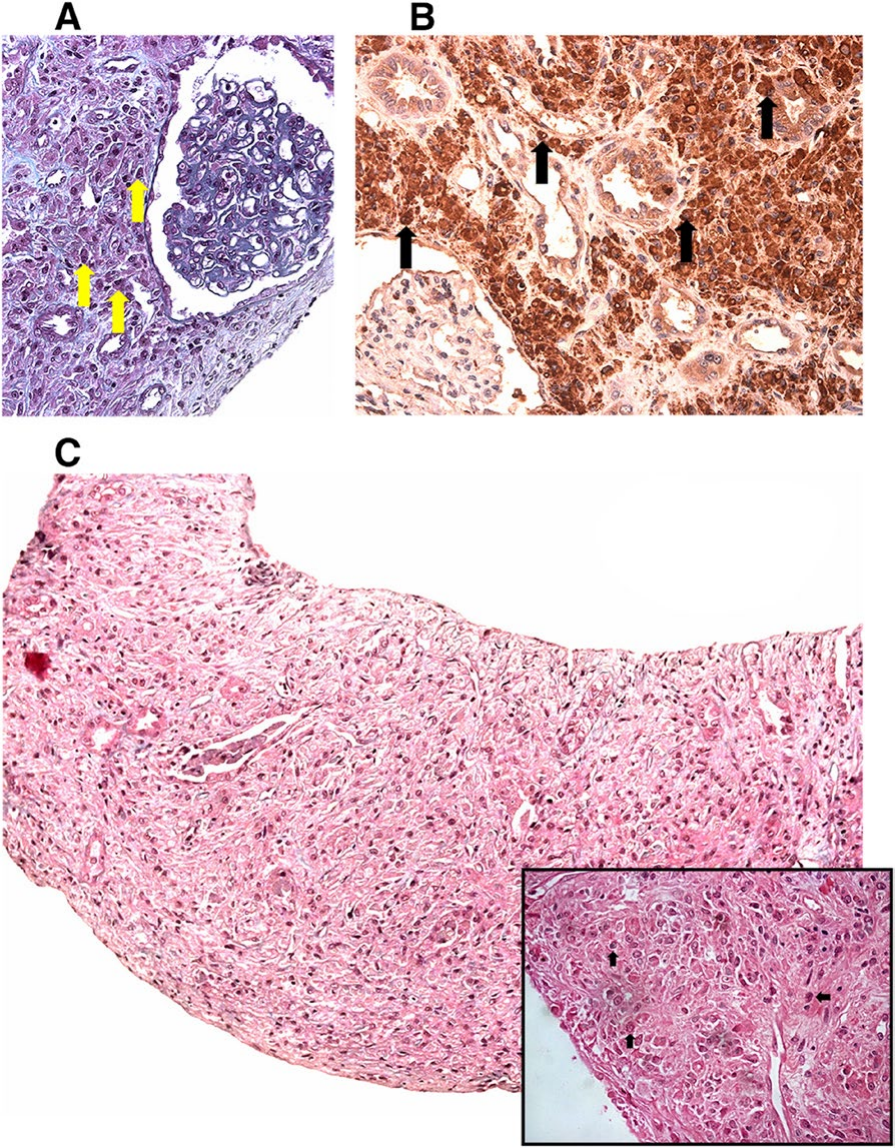
**A** Glomerulus in the patterns of normality and interstitium expanded by numerous mononuclear cells—see arrows (Masson's trichome staining 40 × lens). **B **Mononuclear cells of the interstitium showed positivity for CD68 (histiocyte)—see arrows—and were negative for S100 protein (Immuno-histochemistry. 40 × lens). **C** Interstitium expanded by numerous mononuclear cells—see arrows—(hematoxylin and eosin staining 5 × lens; insert 100 × lens)

A 40-year-old woman with chronic kidney disease (unknown etiology) underwent haemodialysis treatment for eight years and, after that period, she received a kidney transplant from a deceased donor (DDKT). The patient had no family history of nephropathy. Ten years after this DDKT, there was graft loss due to chronic graft changes. She was submitted to haemodialysis for four more years and underwent a second DDKT. In this second transplant, rabbit antithymocyte globulin was used as induction therapy, and sodium mycophenolate, tacrolimus, and prednisone as maintenance immunosuppression. Two months later, she presented with antibody-mediated acute rejection (Banff 2 types I and II) and responded to treatment with prednisolone, plasmapheresis, and intravenous human immunoglobulin, maintaining stable creatinine around 1.6 mg/dL. One year after DDKT, she presented asthenia, myalgia, and a body temperature of 39ºC. Central nervous symptoms were absent. There was no hepato-splenomegaly or cutaneous lesions. Haemoglobin was 6.1 g/dL, MCV: 95 fL, leukocytes: 1.6 × 109/µL, neutrophils: 1.3 × 109/µL, platelets: 143 × 109/µL, reticulocyte count: 112 × 109/L and blood creatinine was 11.8 mg/dL requiring dialysis. The median of haematological parameters and body temperature is represented as the median (range minimum and maximum) during hospitalization (Table 1). The patient was not tested for EBV infection. Blood qPCR (quantitative real-time polymerase chain reaction) for cytomegalovirus (CMV) showed 5219 IU/Ml. Ganciclovir was started at 1.25 mg/kg/day and maintained for 21 days until qPCR became negative. Urine culture revealed > 100,000 cols of Escherichia coli and *Klebsiella pneumoniae*; 5 days later, *Acinetobacter baumanii* and *Klebsiella pneumoniae* grew in the urine (sediment showed 135 leukocytes per higher power field). Thirteen days later, urine culture was negative, and thirty-two days later, urinary sediment showed no leukocytes. Blood culture was positive for oxacillin-resistant *Staphylococcus haemolyticus.* During these episodes of infection, the patient used ceftriaxone, cefepime, amikacin, and meropenem and required regular red blood cell transfusions. Blood counterimmune electrophoresis (fungal polysaccharide antigen search) was positive for *Aspergillus* sp (1/3). A computed tomography scan of the chest and abdomen revealed pulmonary nodules with suspicion of angioinvasive fungal infection and absence of organomegaly. The patient was treated with voriconazole. Fifteen days after the initial presentation, the immunosuppressants were discontinued, leaving only prednisone. The patient had a past medical history of hypertriglyceridemia and hyperferritinemia from the beginning of the second KT (kidney transplant). One year after the second KT, the patient had persistent hyperferritinemia (1,911 ng/mL – 3,268 ng/mL, median 2,093 ng/mL) and hypertriglyceridemia (113 mg/dL—397 mg/dL, median 239 mg/dL). A kidney biopsy (29 days after admission) revealed diffuse histiocytic infiltration (Fig. 1) and was negative for adenovirus, CMV, and Polyoma BK. Mononuclear cells of the interstitium in the kidney biopsy showed positivity for CD68 (histiocyte), and the biopsy was negative for S100 protein which ruled out Langerhans cell histiocytosis (LCH). A bone marrow aspirate smear revealed hemophagocytic figures, whereas bone marrow histology was normal (both procedures were performed 36 days after patient admission). The patient did not receive any other treatment, such as high-dose steroids or IVIG (Intravenous Immunoglobulin). The patient was discharged 40 days later, with haemoglobin 10.4 g/L, evolved with definitive graft loss, and has been on haemodialysis for three years since the onset of the massive histiocytes renal interstitial infiltration.

**Table 1:** Laboratory data from the patient during the 40 days of admission at the hospital

Variable	Median	Range (Min – Max)	Reference value
Temperature (ºC)	36.3	35–39	36–37.8
Haemoglobin (g/dL)	8.45	6.1–10.4	12.4–16.1
Hematocrit (%)	27	18–32	35.4–4.3
WBC (× 10^9^/µL)	2.9	0.6–12.8	4.05–11.84
Neutrophil(× 10^9^/µL)	1.95	0–11.8	1.7–7.2
Platelets (× 10^9^/µL)	157	91–283	203–445
PT	1.03	0.98–1.43	< 1.3
aPTT	1.22	0.96–1.8	< 1.26

Reference values according to the local university hospital laboratory

*WBC* White Blood Cell counts, *PT* prothrombin time, *aPTT* activated partial thromboplastin time

## Discussion and conclusion

Renal interstitial infiltration by histiocytes is rare in KTR patients; it is described in cases like LCH and hemophagocytic lymphohistiocytosis (HLH). HLH results from intense and dysregulated immunological activation of the immune system, which may be triggered by neoplasias, autoimmune disorders, or infections, especially viral infections, such as CMV [3, 4]. The patient presented only 5 out of 8 HLH-2004 diagnostic criteria [2, 4]: fever, elevated ferritin, hypertriglyceridemia, and hemophagocytic figures in the bone marrow aspirate smear. The patient's HLH-probability calculator (HScore) (http://saintantoine.aphp.fr/score/) was 179 [5, 6]. Nearly all patients with HLH have hepatitis, but the patient had normal liver enzymes, bilirubin, albumin, and coagulation parameters (activated partial thromboplastin time, prothrombin time). In adults, ferritin values, characteristic of HLH, are often between 7,000 to 10,000 mg/L, but the patient's maximum ferritin level was 3,268 ng/mL. HLH is a rapidly progressive, life-threatening syndrome of excessive immune activation, and prompt treatment initiation is essential for the survival of affected patients. The patient was not submitted to any specific treatment for HLH. Renal histiocyte activation and infiltration may have been initiated by an immunological mechanism similar to HLH and infectious processes [4]. During an infection, the initial step is the activation of antigen-presenting cells, which promotes the Th1(T helper cells type 1) response to cause the expansion and proliferation of cytotoxic T cells and NKT (Natural killer T cells) in response to the secretion of interleukin (IL-12) and tumour necrosis factor (TNF). In turn, cytotoxic cells release interferon-gamma and granulocyte–macrophage colony growth factor, which lead to the proliferation of histiocytes, infiltration by macrophages, and the production of TNF, IL-1, and IL-6. Several viruses can lead to the activation of T cells and initiate this immune response, as well as bacteria and fungi [4, 7]. The patient had multiple infections, which is relatively common in kidney transplant recipients [8, 9], including CMV, aspergillosis, bacteraemia, and urinary tract infections, which could trigger the immune response. We were not able to find any similar cases in the literature. The present case highlights the occurrence of isolated massive renal interstitial infiltration of histiocytes that does not meet the criteria for HLH or other related pathologies.

## Data Availability

Data supporting the findings of this case report, as well as all data sets generated and analysed during the current study, are available from the corresponding author on reasonable request.

## Abbreviations

AKI: Acute kidney injury
KTR: Kidney transplant recipient
DDKT: Deceased donor kidney transplantation
MCV: Mean corpuscular volume
KT: Kidney transplant
qPCR: Real-time quantitative polymerase chain reaction
CMV: CytomegalovirusS
LCH: Langerhans cell histiocytosis
HLH: Haemophagocytic lymphohistiocytosis

## References

[1] Camargo-Salamanca A, Garcia-Lopez A, Patino-Jaramillo N, Giron-Luque F (2020). Acute kidney injury in hospitalized kidney transplant recipients. Transplant Proc.

[2] Filiponi TC, Requiäo-Moura  LR, Tonato  EJ , Carvalho de Matos  AC, Silva-Filho  APE, de Souza Durão Junior  M (2015). Hospital admission following acute kidney injury in kidney transplant recipients is associated with a negative impact on graft function after 1-year. PLoS One.

[3] Henter JI, Horne A, Arico M (2007). HLH-2004: diagnostic and therapeutic guidelines for hemophagocytic lymphohistiocytosis. Pediatr Blood Cancer.

[4] Karras A (2009). What nephrologists need to know about hemophagocytic syndrome. Nat Rev Nephrol.

[5] La Rosee P, Horne A, Hines M (2019). Recommendations for the management of hemophagocytic lymphohistiocytosis in adults. Blood.

[6] Fardet L, Galicier L, Lambotte O (2014). Development and validation of the HScore, a score for the diagnosis of reactive hemophagocytic syndrome. Arthritis Rheumatol.

[7] Delves PJ, Roitt IM (2000). The immune system. first of two parts. N Engl J Med.

[8] Fishman JA (2017). Infection in organ transplantation. Am J Transplant.

[9] Sommerer C, Schröter I, Gruneberg K, Schindler D, Behnisch R, Morath C, Renders L, Heemann U, Schnitzler P, Melk A, Penna AD, Nadalin S, Heeg K, Meuer S, Zeier M, Giese T (2022). Transplant cohort of the German center for infection research (DZIF Transplant Cohort) consortium. Open Forum Infect Dis.

